# Fluid Flow and Stress Field During Laser Cladding-Based Surface Repair of Aluminum Alloy: Multi-Track Simulation

**DOI:** 10.3390/ma18071603

**Published:** 2025-04-02

**Authors:** Quan Wu, Haiping Chu, Zhongkui Liu, Lihang Yang, Xiaosong Zhou, Yinfeng He, Yi Nie

**Affiliations:** 1 School of Mechanical and Electrical Engineering, Guizhou Normal University, Guiyang 550001, China; 2 Nottingham Ningbo China Beacons of Excellence Research and Innovation Institute, University of Nottingham Ningbo China, Ningbo 315100, China; 3 Faculty of Science and Engineering, University of Nottingham Ningbo China, Ningbo 315100, China; 4 Faculty of Engineering, University of Nottingham, Nottingham NG7 2RD, UK

**Keywords:** laser cladding, surface repair, aluminum alloy, melt flow, stress analysis, multi-track, defect formation

## Abstract

Laser cladding (LC) is a promising technique for repairing aluminum alloy components, yet challenges like cracks and uneven surfaces persist due to unstable melt flow and thermal stress. This study employs both fluid flow and stress field models to investigate multi-track LC repair mechanisms. Using a finite volume method (FVM), the dynamic evolution of the molten pool was quantified, revealing a maximum flow velocity of 0.2 m/s, a depth of 0.7 mm, and a width of 4 mm under optimized parameters (1600 W laser power, 600 mm/min scan speed). The model also identified that surface flaws between 300 and 900 μm were fully melted and repaired by a current or adjacent track. Finite element analysis (FEA) showed that multi-layer cladding induced a cumulative thermal stress exceeding 1300 MPa at interlayer interfaces, necessitating ≥ 3 s cooling intervals to mitigate cracking risks. These findings provide critical insights into process optimization, demonstrating that adjusting laser power and scan speed can control molten pool stability and reduce residual stress, thus improving repair quality for aluminum alloys.

## 1. Introduction

Aluminum alloys are extensively employed in the aerospace and automotive industries owing to their high specific strength, corrosion resistance, and lightweight nature. Nevertheless, localized surface damage caused by wear, corrosion, or fatigue during prolonged service critically degrades their performance and lifespan. While surface repair presents a time- and cost-efficient alternative to full component replacement, conventional techniques (e.g., arc welding) often induce substrate deformation and residual stresses due to excessive heat input. In contrast, laser cladding (LC)—also termed laser-directed energy deposition (LEDE)—utilizes a high-energy laser beam to metallurgically bond a delivered powder/wire with the substrate under low heat input [1,2]. This enables precision repair with minimal thermal distortion, a narrow heat-affected zone, and dense microstructures, positioning LC as a promising strategy for aluminum alloy restoration [3,4].

However, the unique physical properties of aluminum alloys (such as high thermal conductivity, low melting point, and high reflectivity to lasers) and their complex multi-physics field behavior during the repair process pose significant challenges to process optimization and defect control. While costly experimental methods like high-speed imaging partially capture molten pool behavior, computational simulations offer granular insights into localized thermal gradients, fluid velocities, and stress fields. The validated multi-physics model herein enables a quantitative analysis of laser–powder–substrate interactions, identifies defect formation thresholds, and guides intelligent parameter optimization to advance sustainable repair strategies.

The two key factors that must be considered when establishing such a model are the molten flow of the metal alloy and the stress field during the melting and cooling processes. The only numerical study on molten flow during LC-based surface repair was conducted by Zhou et al., who used a DEM-FVM model to study single-track LC, but their assumption of a pre-defined powder bed neglected powder momentum and particle–molten pool interactions, limiting its predictive accuracy in dynamic processes [5]. In more recent studies, numerical research on LC-based additive manufacturing has become increasingly popular, focusing on the dynamic behavior, physical size, and shape of the delivered powder stream. These studies typically treat the powder particles as individual Lagrangian particles using the Discrete Phase Method (DPM) [6,7]. While such models can illustrate the shape and flow behavior of the molten pool, they have a limited capacity to fully capture laser–particle interactions and momentum transfer to the molten pool. To better quantify the mass, momentum, and energy introduced by the powder stream, Sun et al. developed a powder source model that accurately predicts the deposited track dimensions (width, height, and dilution depth), yet stress evolution and defect formation mechanisms were not addressed [8].

The stress field during the LC process are normally simulated using the finite element method. Stress field simulation is crucial for understanding the formation of residual stress and the localized deformation it causes [9,10]. Most studies applied a transient direct coupled thermo-mechanical model in which the temperature distribution was calculated and imported into a following structural analysis to calculate local stress and displacement. Gao et al. established a three-dimensional numerical single-track processing prediction model by the element birth and death method [11]. This innovative model enabled the computation of the transient temperature field and the geometry of the cladding simultaneously, providing a comprehensive understanding of the temperature distribution law in molten pools and the varying principle of the forming parts’ cross section sizes during the LC process. Tamanna et al. developed a three-dimensional finite element transient thermo-mechanical model to predict the thermal profile and residual stress distribution for the LC-based surface repair of a Ti6Al4V alloy [12]. A moving heat source was incorporated into the model, accounting for the dynamic nature of the repair process and enhancing the accuracy of the predictions.

The existing studies either focus solely on single-track molten pool behavior (neglecting intertrack interactions) or isolated stress analysis without dynamic melt flow consideration, limiting their ability to predict defect formation in multi-track repairs. To address these gaps, a multi-physics model that integrates fluid flow and a stress field model for multi-track LC repair was developed. The model incorporates powder momentum and energy transfer, Marangoni-driven melt dynamics, and element birth/death for progressive material deposition. This approach quantifies molten pool stability, defect repair efficiency, and stress accumulation, providing insights for optimizing process parameters to minimize cracks and improve repair quality.

## 2. Fluid Flow Modeling During LC-Based Surface Repair Process

### 2.1. Mathematical Modeling of the Fluid Flow

**Figure 1:**
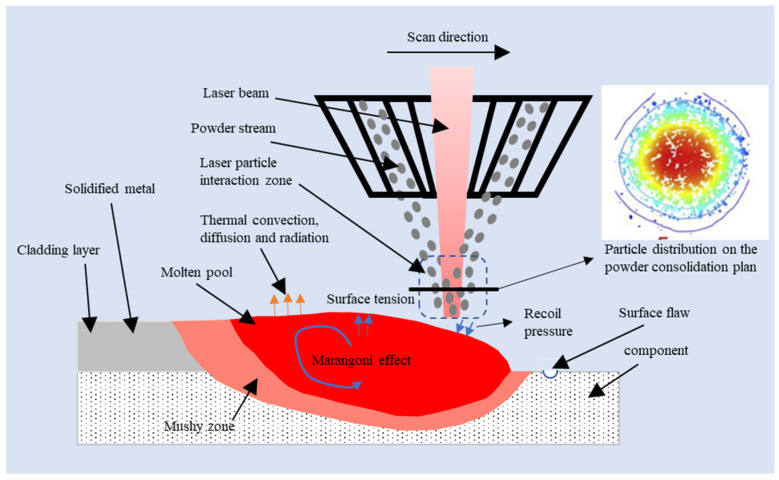
Schematic diagram of fluid flow during LC-based surface repair process.

The physical phenomena involved in the LC-based repair process start with the laser radiation (e.g., a Gaussian laser beam) of the injected co-axial metal powder and the rapid melting and formation of a molten pool on the component’s surface, as shown in Figure 1. Heat dissipation due to convection, diffusion, and radiation happens on the surface of the molten pool simultaneously. Surface tension is exerted on the interface of the molten pool and surrounding gas (e.g., a protection argon gas). Within the molten pool, the Marangoni effect due to the temperature gradient, drag force in the mushy zone, recoil pressure due to vaporization of metal material, and internal gravity and buoyancy force drives the molten metal flowing around and filling the surface flaws on the component. The molten pool rapidly cools and solidifies, forming the deposited track after the laser scans the area. The stability of the molten pool during the single- and multi-track LC process will directly affect the surface quality and internal structure of the repaired surface.

Before establishing a numerical model of the molten pool flow process, it is necessary to reasonably simplify the complex physical process to ensure the effectiveness of the calculation. To ensure the efficiency of the numerical simulation, this model considers incompressible Newtonian fluid laminar flow [13], ignores the mass loss caused by metal vaporization, does not consider the impact of the volume change caused by the metal density change, and ignores the oxidation of metal materials [5].

#### 2.1.1. Governing Equations

The formation, flow, and cooling of the molten pool during the LC process involve multi-phase fluid flow. The whole process is governed by the following mass, momentum, and energy conservation equations:

(1) $$\frac{\partial \bar{\rho }}{\partial t}+\nabla \cdot \left(\bar{\rho }u\right)=\dot{M}$$

(2) $$\frac{\partial (\bar{\rho }u)}{\partial t}+\nabla \cdot \left(\bar{\rho }uu\right)=-\nabla P+\bar{\mu }\nabla^{2}u+S_{m}$$

(3) $$\frac{\partial \bar{\rho }\bar{c}T}{\partial t}+\nabla \cdot \left(\bar{\rho }u\bar{c}T\right)=\nabla \cdot \left(\bar{k}\nabla T\right)+S_{e}$$

where t represents time, u is velocity vector, P is pressure, and T is temperature. $\dot{M}$, $S_{m}$, and $S_{e}$ are the mass, momentum, and energy source terms, respectively. $\bar{\rho }$, $\bar{\mu }$, $\bar{c}$, and $\bar{k}$ are, respectively, the uniform density, viscosity, specific heart, and conductivity of the mesh cell, which are related to the pure-phase material properties as

(4) $$\bar{\rho }=\alpha_{1}\rho_{1}+\alpha_{2}\rho_{2}$$

(5) $$\bar{\mu }=\alpha_{1}\mu_{1}+\alpha_{2}\mu_{2}$$

(6) $$\bar{c}=\left\{\begin{array}{c} \alpha_{2}\left(c_{2}+\frac{L_{f}}{T_{L}-T_{S}}\right)+\alpha_{1}c_{1}\;\;T_{L}<T<T_{S}\\ \alpha_{1}c_{1}+\alpha_{2}c_{2}\;\;\;\;T\geq \;T_{L}\;or\;T\leq T_{S} \end{array}\right.$$

(7) $$\bar{k}=\alpha_{1}k_{1}+\alpha_{2}k_{2}$$

where $\rho_{1}$, $\mu_{1}$, $c_{1}$, and $k_{1}$ are the density, viscosity, specific heart, and conductivity of the gas phase, while $\rho_{2}$, $\mu_{2}$, $c_{2}$, and $k_{2}$ are the density, viscosity, specific heart, and conductivity of the metal phase. $L_{f}$ is the latent heat of fusion, and $T_{L}$ and $T_{S}$ are the liquidus and solidus temperatures of the metal phase.

A multi-phase fluid model was employed, and the Volume of Fluid (VOF) method was used to track the interface between the metal and gas phases. The gas and metal volume fraction in a mesh cell is defined as $\alpha_{1}$ and $\alpha_{2}$, respectively. $\alpha_{2}=1$ indicates that the cell is occupied by the entire metal, while $\alpha_{2}=0$ indicates that this cell contains no metal. If $0<\alpha_{2}<1$, it indicates that the cell is a mixture of both gas and metal. The conservation of the metal volume fraction can be derived as

(8) $$\frac{\partial \alpha_{2}}{\partial t}+\nabla \cdot \left(\alpha_{2}u\right)=\dot{\alpha_{2}}$$

(9) $$\alpha_{1}+\alpha_{2}=1$$

According to the metal volume fraction, the unit normal vector n and surface curvature at the metal–gas interface can be derived as

(10) $$n=\frac{\nabla \alpha_{2}}{\left|\nabla \alpha_{2}\right|}$$

(11) κ=−∇·n

The finite volume method (FVM) was employed to solve the governing equations for mass, momentum, and energy conservation due to its direct discretization in physical space and versatile applicability to both structured and unstructured grid systems.

#### 2.1.2. Mass Source Terms

**Figure 2:**
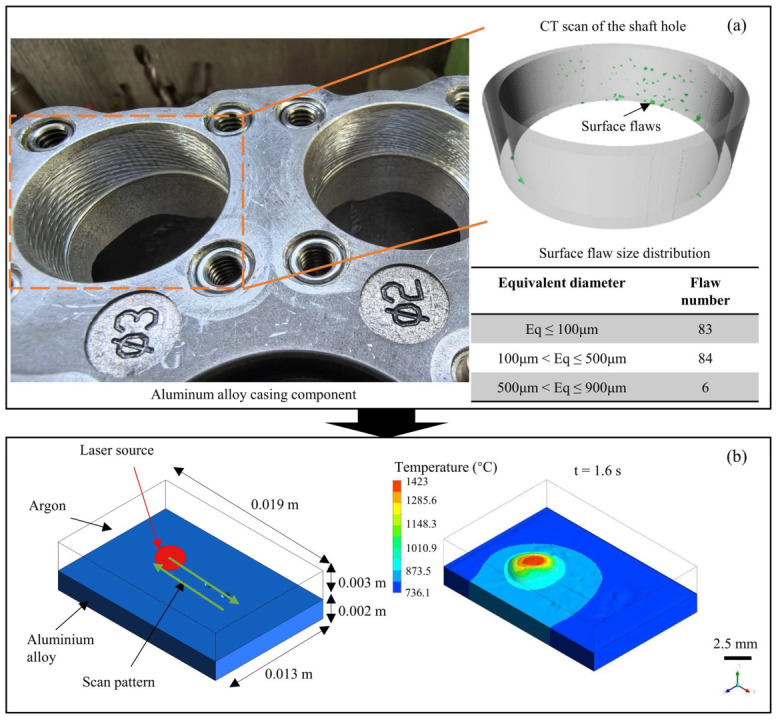
Technology roadmap for numerical simulation of fluid flow during LC-based surface repair process: (**a**) CT scan of surface flaws on aluminum alloy casing component and [5] (**b**) numerical model and intended simulation results of molten pool during double deposition track.

The mass source in the molten pool is introduced by the powder stream. With a small powder beam focal length, high laser power, and long laser–powder interaction time in the powder-based LC process, enough laser energy could be transferred to the powder particles to the allow their temperature to reach the melting point before entering the molten pool [8]. Therefore, the powder particles can be assumed as droplets ejected perpendicular to the molten pool, and the particles are at the melting temperature. The particle intensity distribution on the horizontal plane of the powder stream can be assumed as a Gaussian distribution, as shown in Figure 2. The mass source is termed $\dot{M}$ and, affected by the powder stream, is then represented as follows [14]:

(12) $$\dot{M}=\frac{2PFR\cdot \eta_{p}}{\pi r_{p}^{2}}exp\left(-2\frac{{{\left(x-x_{0}-vt\right)}^{2}+\left(z-z_{0}\right)}^{2}}{r_{p}^{2}}\right)\left|\nabla \alpha_{2}\right|\frac{2\bar{\rho }}{\rho_{1}+\rho_{2}}$$

where $\eta_{p}$ is the utilization efficiency of the powder particles (typically as 90%), PFR is the mass flow rate of the powder stream, and $r_{p}$ is the diameter of the powder consolidation plane. The term $\frac{2\bar{\rho }}{\rho_{1}+\rho_{2}}$ is used to improve the numerical stability of the model. x and z are the coordinate system. v is the laser scanning speed, which means the powder stream moves together with the laser head in the x direction. $x_{0}$ and $z_{0}$ are the starting point of the laser head.

#### 2.1.3. Momentum Source Terms

The momentum sources, which include the powder impingement $S_{pi}$, surface tension $S_{st}$, recoil pressure $S_{rp}$ at the melt-free surface, Marangoni effect $S_{me}$, gravity force $S_{gf}$, buoyancy force $S_{bf}$ inside the molten pool, and mushy zone drag force $S_{mz}$ at the liquid and solid interface, can be expressed as

(13) $$S_{m}=S_{pi}+S_{st}+S_{rp}+S_{me}+S_{gf}+S_{bf}+S_{mz}$$

The powder impingement $S_{pi}$ can be written as [8]

(14) $$S_{pi}=\left[\frac{3\mu C_{D}R_{e}}{4{\bar{\rho }}_{p}{\bar{d}}_{p}^{2}}\left(U_{p}-U\right)+\frac{g\left({\bar{\rho }}_{p}-\rho \right)}{{\bar{\rho }}_{p}}\right]∆t\dot{M}\left|\nabla \alpha_{2}\right|\frac{2\bar{\rho }}{\rho_{1}+\rho_{2}}$$

where $U_{p}$ is the flow velocity of the shielding protection gas supplied together with the powder stream. ∆t is the numerical time interval, and $C_{D}$ is the drag coefficient, which can be calculated as

(15) $$C_{D}=\frac{24}{R_{e}}\;when\;R_{e}\leq 1$$

(16) $$C_{D}=\frac{24}{R_{e}}\left(1+0.15{R_{e}}^{0.687}\right)\;when\;1<R_{e}\leq 1000$$

(17) $$C_{D}=0.44\;when\;R_{e}>1000$$

among which the Reynolds number $R_{e}$ is expressed as

(18) $$R_{e}=\frac{\rho d_{p}\left|U-U_{p}\right|}{\mu }$$

The surface tension $S_{st}$ is normal to the free surface and can be obtained by the continuous surface force (CSF) model as [15]

(19) $$S_{st}=\sigma \kappa n\left|\nabla \alpha_{2}\right|\frac{2\bar{\rho }}{\rho_{1}+\rho_{2}}$$

where σ is the surface tension coefficient. The recoil pressure $S_{rp}$ acts perpendicularly to the local free surface and is a function of the melted surface temperature [16]

(20) $$S_{rp}=0.54P_{a}exp\left(\frac{L_{v}M\left(T-T_{V}\right)}{RTT_{V}}\right)n\left|\nabla \alpha_{2}\right|\frac{2\bar{\rho }}{\rho_{1}+\rho_{2}}$$

where $P_{a}$ is the atmosphere pressure, $L_{v}$ is the latent heat of evaporation, M is the molar mass of the metal material, R is the universal gas constant, and $T_{V}$ is the evaporation temperature of the metal material. The Marangoni effect $S_{me}$, induced by the dependence of the surface tension coefficient on the temperature $\frac{d\sigma }{dT}$, can be expressed as [17]

(21) $$S_{me}=\frac{d\sigma }{dT}[\nabla T-\left(n\cdot \nabla T\right)n]\left|\nabla \alpha_{2}\right|\frac{2\bar{\rho }}{\rho_{1}+\rho_{2}}$$

The gravity force $S_{gf}$ and buoyancy force $S_{bf}$ are in the downward direction and can be written as [17]

(22) $$S_{gf}=\bar{\rho }g$$

(23) $$S_{b}=\bar{\rho }g\bar{\alpha }(T-T_{ref})$$

where g is the gravity acceleration vector, $\bar{\alpha }$ is the thermal expansion coefficient, and $T_{ref}$ is the reference temperature (typically set as the liquidus temperature of the metal material). The mushy zone drag force $S_{mz},$ induced by phase transformation, is expressed as [16]

(24) $$S_{mz}=-C\left[\frac{{\left(1-f_{l}\right)}^{2}}{{f_{l}}^{3}+C_{m}}\right]u$$

(25) $$f_{l}=\left\{\begin{array}{c} 0\;\;\;if\;T<T_{s}\\ \frac{T-T_{S}}{T_{L}-T_{S}}\;\;if\;T_{S}\leq T\leq T_{L}\\ 1\;\;\;if\;T>T_{L} \end{array}\right.$$

where C is a constant whose value should be large enough to ensure that the velocity drops to zero when the local area is completely solidified. Typically, it is set to 10^5^ or larger. $f_{l}$ is the liquid fraction of the metal phase. $C_{m}$ is a custom small value used to avoid singularities in the mushy regions during the drag force calculation.

#### 2.1.4. Energy Source Terms

The energy source during the LC process includes the laser heat source term $S_{l}$, convection heat loss term $S_{c}$, radiation heat loss term $S_{r}$, and evaporation heat loss term $S_{e}$, and the heat source induced by the powder stream can be expressed as

(26) $$S_{e}=S_{l}+S_{c}+S_{r}+S_{e}+S_{p}$$

A Gaussian laser beam was used as the heat source, which was applied to the metal–gas interface. The laser heat source term $S_{l}$ can be rewritten volumetrically with a continuum surface force (CSF) model as [17]

(27) $$S_{l}=\frac{2\eta P_{laser}}{\pi r^{2}}exp\left(-2\frac{{\left(x-x_{0}-vt\right)}^{2}+{\left(z-z_{0}\right)}^{2}}{r^{2}}\right)\left|\nabla \alpha_{2}\right|\frac{2\bar{\rho }\bar{c}}{\rho_{1}c_{1}+\rho_{2}c_{2}}$$

where η is the laser absorption coefficient, $P_{laser}$ is the laser power, r is the laser beam diameter at the metal–gas interface, and the term $\frac{2\bar{\rho }\bar{c}}{\rho_{1}c_{1}+\rho_{2}c_{2}}$ is used to improve the numerical stability of the model at the metal–gas interface. The volumetric heat loss terms due to convection, radiation, and evaporation on the metal–gas interface are expressed as [16,18]

(28) $$S_{c}=h_{c}\left(T-T_{a}\right)\left|\nabla \alpha_{2}\right|\frac{2\bar{\rho }\bar{c}}{\rho_{1}c_{1}+\rho_{2}c_{2}}$$

(29) $$S_{r}=k_{B}\varepsilon \left(T^{4}-T_{a}^{4}\right)\left|\nabla \alpha_{2}\right|\frac{2\bar{\rho }\bar{c}}{\rho_{1}c_{1}+\rho_{2}c_{2}}$$

(30) $$S_{e}=-\varphi \frac{L_{v}M}{\sqrt{2\pi MRT}}P_{a}exp\left[\frac{L_{v}M\left(T-T_{v}\right)}{RTT_{v}}\right]\left|\nabla \alpha_{2}\right|\frac{2\bar{\rho }\bar{c}}{\rho_{1}c_{1}+\rho_{2}c_{2}}$$

where $h_{c}$ is the convective coefficient, ε is the radiation emissivity, $k_{B}$ is the Stefan–Boltzmann constant, $T_{a}$ is the ambient temperature, φ is the evaporation coefficient, and $L_{v}$ is the latent heat of evaporation. The volumetric heat source introduced by the assumed powder droplet can be expressed as

(31) $$S_{p}=C_{p}∆T_{p}\dot{M}\left|\nabla \alpha_{2}\right|\frac{2\bar{\rho }\bar{c}}{\rho_{1}c_{1}+\rho_{2}c_{2}}$$

where $C_{p}$ is the specific heat of the powder particles and $∆T_{p}$ is the temperature change in the particles within each numerical interval.

### 2.2. Numerical Implementation of the Fluid Flow Simulation

This simulation utilized ANSYS 2022 R1 Fluent to conduct numerical simulation on the above physical equations in the LC-based surface pair process, as shown in Figure 2. Considering the CT scanning data of the aluminum alloy casing component obtained using an engineering-grade CT scanner (model nanoVoxel-4000 from Tianjin Sanying Precision Instrument Co., Ltd. located in Tianjin, China) with a 2 μm voxel resolution, a local region on the shaft hole was selected and simplified into a thin-walled flat plate with dimensions of 19 × 13 × 2 mm. Surface flaws were reconstructed on the plate from the scan data, with equivalent diameters ranging from 0.3 to 0.9 mm (as shown in Figure 2a). The flaws were distributed along the first deposition track at 2 mm intervals, greater than the laser beam diameter. To capture the free surface of the molten pool, the shielding gas of argon around the top of the metal plate was also included in the simulation model. The zig-zag laser scanning strategy was employed, and the front and back deposition tracks were considered. The laser irradiated the surface of the metal material along the vertical direction (in the negative direction of the y-axis), scanned ahead along the positive direction of the x-axis from the origin at first, and scanned in the opposite direction along the negative direction of the x-axis after the first track. The gravitational acceleration of 9.81 m/s in the negative direction of the y-axis was considered.

**Figure 3:**
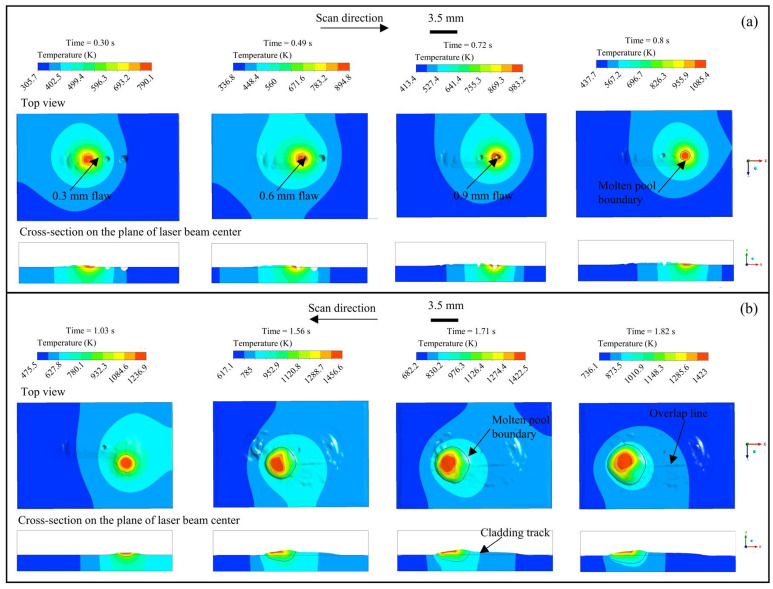
Evolution of the morphology and temperature field of the molten pool at various time points: (**a**) first single track along positive x-axis and (**b**) second track along negative x-axis.

The numerical simulation of the fluid flow in the molten pool involved the meshing of the computational domain, material property-setting, boundary condition-setting, process parameter-setting, and numerical calculation control. As shown in Figure 3, since a great temperature gradient exists at the metal–gas interface where the molten pool is generated, the mesh was refined there. Considering a minimum surface flaw size of 0.3 mm, the minimum mesh size was set to 0.1 mm. The maximum mesh size was set to 0.4 mm considering the maximum geometric feature size of 19 mm. The computation domain was discretized using hexagonal elements with a total mesh number of 617,500. The constant solidus and liquidus material properties of the AlSi10 Mg were considered considering computational efficiency, as shown in Table 1. The viscosity of 100 kg/(m s) was assigned to the aluminum alloy to represent its solidus status. Other related thermodynamic parameters include the energy absorption coefficient of the aluminum alloy to the laser beam of 0.35 [19], thermal convective coefficient of 82 W/(m^2^ K) [20], thermal radiation emissivity of 0.4 [20], thermal evaporation coefficient of 0.82 [13], universal gas constant of 8.1344 J/(mol K), Stefan–Boltzmann constant of 5.67 × 10^8^ W/(m^2^ K^4^), and ambient pressure of 101,325 Pa. The material properties of the argon gas were obtained from the ANSYS Fluent database, as shown in Table 2.

The processing parameters during the LC-based surface repair process included a laser beam diameter of 3.5 mm, laser power of 1600 W, scanning speed of 600 mm/min, hatch spacing of 2 mm, powder stream diameter of 3.5 mm, powder stream mass flow rate of 3 × 10^−5^ Kg/s, shielding argon gas flow rate of 8.3 × 10^−5^ m^3^/s, and average particle size of the powder stream of 80 µm. The top and side surfaces of the sub-domain containing the argon phase were defined as pressure outlets that open to the ambient atmosphere with zero gauge pressure and ambient temperature. The side and bottom surfaces of the sub-domain containing the aluminum alloy substrate were set as non-slip walls, with their thermal convection and radiation expressed in Equations (28) and (29) [21]. During the numerical calculation, the mass, momentum, and energy source terms were loaded into the calculation domain using a User-Defined Function (UDF). The pressure was discretized using the PRESTO method, the momentum was discretized using the second-order upwind method, the energy was discretized using the second-order upwind method, and the velocity–pressure coupling was processed using the PISO method. The dynamic time step was applied with the initial time step set as 1 × 10^−7^ s and a total time of 1.8 s. The simulation was finished after 48 calculation hours.

**Table 1:** Physical properties of AlSi10 Mg applied in numerical simulation.

Name	Value
Solidus density (kg/m^3^)	2719 [5]
Liquidus density (kg/m^3^)	2490 [5]
Solidus specific heat (J/(kg K))	906 [22]
Liquidus specific heat (J/(kg K))	1220 [22]
Solidus thermal conductivity (W/(m K))	202.4 [22]
Liquidus thermal conductivity (W/(m K))	61 [22]
Dynamic viscosity (kg/(m s))	0.004 [20]
Molecular mass (kg/kmol)	26.982
Reference temperature (K)	298.15
Latent heat of melting (J/kg)	383,000 [22]
Latent heat of vaporization (J/kg)	10,870,000 [22]
Solidus temperature (K)	890 [22]
Liquidus temperature (K)	929 [22]
Surface tension coefficient (N/m)	0.914 − 0.35 × 10^−3^(*T* − 890) [23]

### 2.3. Results and Analysis

The evolution of the morphology and temperature field of the molten pool at different time instants during the first cladding track was investigated, as shown in Figure 3a. The computational results indicate that the surface flaws of 300 and 600 µm were not successfully cladded, owing to the fact that the metal material failed to melt at the commencement of the cladding process, and consequently, there was no molten pool flow. In contrast, the 900 µm surface flaw was successfully cladded, and the resultant surface was smooth. This was attributed to the generation of the molten pool, which facilitated material flow and enabled the repair of the defect. Hence, the energy input at the starting position is inadequate for surface repair. It is recommended that the repair operation should be initiated after 0.7 s, when the molten pool is initiated.

The evolution of the morphology and temperature field of the molten pool at various time points during the second cladding track was analyzed, as shown in Figure 3b. The calculations revealed that the molten pool exhibited the highest temperature at the center of the laser beam, which progressively decreased along both the surface and depth directions under a significant gradient. Under the influence of surface tension and powder stream pressure, the front end of the molten pool was lower than the rear end. As time increased, the molten pool expanded in both width and depth and achieved stability at 1.7 s. The material at the tail of the molten pool cooled and contracted, resulting in the formation of a flat cladding track. The surface flaws of 300 and 600 µm were successfully repaired via the adjacent second cladding track, suggesting that the melt paths interacted with each other and that the current hatch spacing was appropriate. This indicates that the adjacent cladding tracks interact greatly with each other, and an obvious overlap line was observed between them.

**Figure 4:**
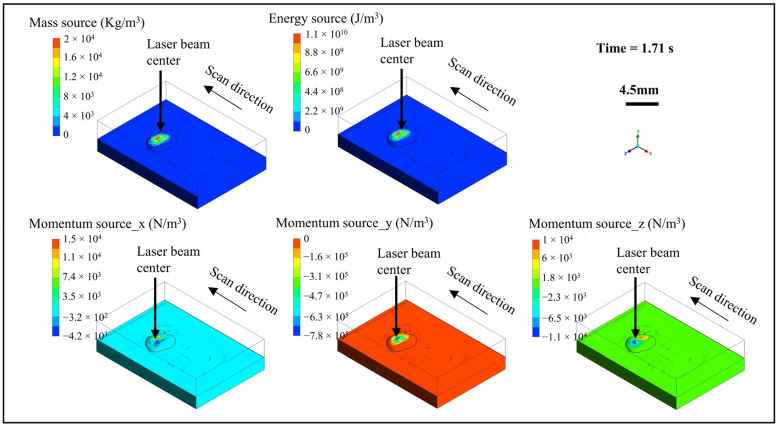
Mass, energy, and momentum source terms introduced by powder streams.

As shown in Figure 4, further analysis on the influence of the powder stream on the molten pool flow reveals that the powder stream increases the mass of the molten pool, thereby forming a cladding layer. The mass flow rate of the powder stream determines the height of the cladding layer. The powder steam also increases the energy of the molten pool. The maximum value of the volumetric energy source term is 1.11 × 10¹⁰ W/m³, while the maximum volumetric energy source introduced by the laser is 4.17 × 10¹¹ W/m³. This indicates that the energy introduced by the powder stream is one order of magnitude smaller than the laser energy. The powder stream brings a momentum source perpendicular and inward to the surface and causes the molten pool to be squeezed downward, resulting in the height of the molten pool head being lower than that of the tail. The volumetric flow rate of the shielding argon gas in the powder beam mainly affects the momentum source introduced by the powder stream.

**Figure 5:**
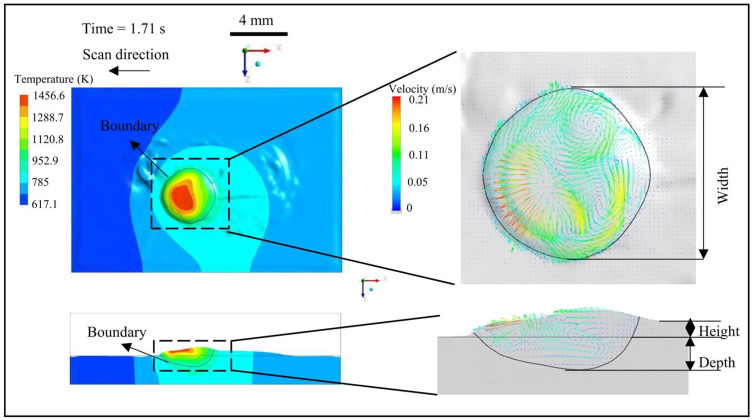
Morphology and flow character of the stable molten pool at time = 1.71 s.

A detailed analysis on the morphology and flow characteristics of the stable molten pool was conducted, as shown in Figure 5. The results show that the molten pool was mainly driven by Marangoni stress to repair the surface flaws of the component. On the surface of the molten pool, the flow occurred from the center to the surroundings, while at the bottom of the molten pool, the flow returned back to the center, forming an eddy flow that drove the surrounding materials into the molten pool. The eddy flow velocity at the front end of the molten pool was high, with a maximum flow velocity of 0.2 m/s. The calculated depth of the molten channel was 0.7 mm, which indicates that the flaws exceeding the depth could probably not be repaired. The width of the molten pool was 4 mm, which was larger than the laser beam diameter, indicating a high heat-affected zone. The height of the molten pool was 0.3 mm, which was determined by the powder stream mass flow rate. The calculated molten pool morphology indicates that the current process parameters can form a stable molten pool sufficient enough for surface repair. This aligns with a prior study [24], which reported that stable molten pools require a balance between laser energy input and thermal dissipation, minimizing both incomplete fusion and excessive dilution.

## 3. Stress Field Simulation During LC-Based Surface Repair Process

**Figure 6:**
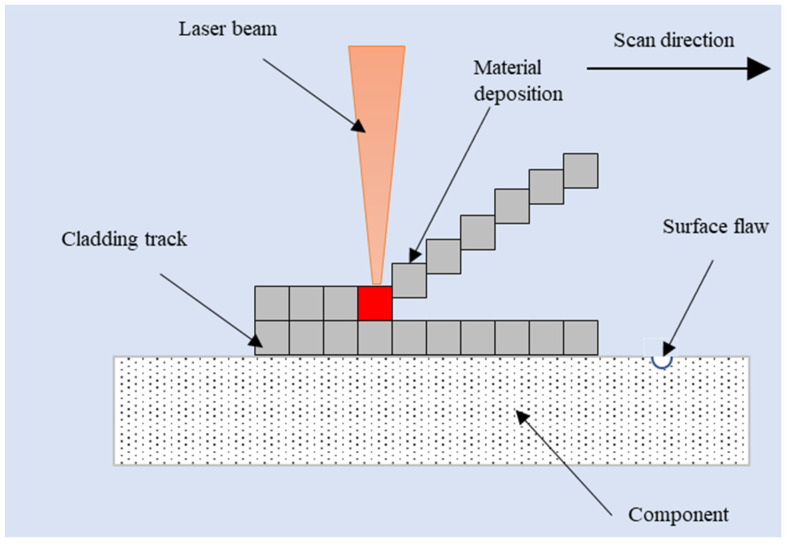
Schematic diagram of stress field during LC-based surface repair process.

Different to the above fluid flow analysis, a finite element model for the stress field during the LC-based surface repair process is separately established. The powder stream is assumed as a solid material that is deposited on the component track by track and layer by layer under the radiation of an accompanying laser heat source, as shown in Figure 6. Heat conduction occurs within the material, causing the temperature to rise and fall rapidly. This repeated heating and cooling leads to a significant temperature gradient, thermal stresses, and deformations within the metal material.

### 3.1. Mathematical Models of the Stress Field

To calculate the stress and deformation fields during the LC-based surface repair process, a direction-coupled thermal–structural analysis will be performed in this paper. The temperature distribution due to the laser beam heat flux is first calculated by thermal analysis. The stress and strain due to expansion and consolidation by temperature change will be calculated by structural analysis then. The governing equations of the process are as follows [24]:

(32) $$\frac{\partial \rho cT}{\partial t}=\nabla \cdot \left(k\nabla T\right)+Q$$

(33) $$∆\sigma_{ij}=D_{ijlm}∆\varepsilon_{ij}^{e}$$

where T is the current temperature, Q is the heat flux, t is time, ρ is the temperature dependent density, c is the temperature-dependent specific heat, and k is the temperature-dependent thermal conductivity. $∆\sigma_{ij}$ is the stress increment, which is related to the elastic stiffness tensor $D_{ijlm}$ and elastic strain $∆\varepsilon_{ij}^{e}$. The elastic stiffness tensor can be express according to Hook’s law as

(34) $$D_{ijlm}=\frac{E}{1+v}\left[\frac{1}{2}\left(\delta_{il}\delta_{jm}+\delta_{lm}\delta_{ij}\right)+\frac{v}{1-2v}\delta_{ij}\delta_{lm}\right]$$

where E is Young’s modulus, v is Poisson’s ratio, and δ is the Dirac function. The elastic strain is represented as

(35) $$∆\varepsilon_{ij}^{e}=∆\varepsilon_{ij}^{tol}-∆\varepsilon_{ij}^{th}-∆\varepsilon_{ij}^{p}$$

where $∆\varepsilon_{ij}^{tol}$ is the total strain increment, $∆\varepsilon_{ij}^{th}$ is the thermal strain increment, and $∆\varepsilon_{ij}^{p}$ is the plastic strain increment. The plastic strain increment can be written as

(36) $$∆\varepsilon_{ij}^{th}=\alpha \delta_{lm}∆T$$

where α and ∆T are the thermal expansion coefficient and temperature increment, respectively.

### 3.2. Numerical Implementation of the Stress Field Simulation

**Figure 7:**
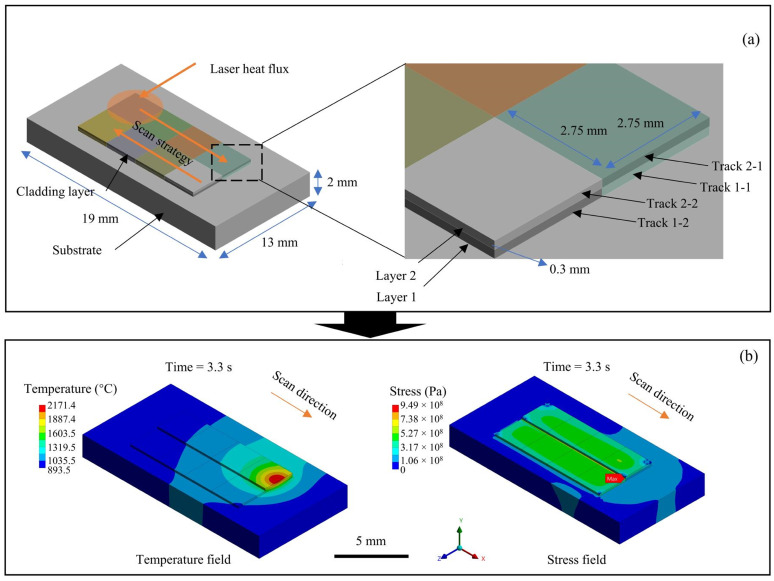
Technology roadmap for numerical simulation of stress field during LC-based surface repair process: (**a**) geometry and finite element model and (**b**) intended simulation results during double-layer deposition.

As shown in Figure 7, the coupled thermal–structural numerical simulation of the LC-based surface repair process still selects the local position of the part and simplifies it into a thin-walled flat plate. The size of the flat plate part remains consistent with the fluid simulation at 19 × 13 × 2 mm. The actual metal material is assumed to be formed from several finite elements, which are clad one by one on the surface of the part to form multi-tracks and layers. Considering two claddings within the one layer and two interlayer claddings, the tracks in layer 1 are named 1-1 and 1-2, and the tracks in layer 2 are named 2-1 and 2-2. The element birth and death method is employed to simulate the progressive material deposition during laser cladding. By selectively activating (“birthing”) elements in alignment with the cladding path and laser heat source motion, this approach replicates the point-by-point, track-by-track, and layer-by-layer additive process. Inactive elements are initially assigned near-zero stiffness to avoid spurious stresses, while their material properties are gradually restored upon activation to mimic molten powder material integration. An accompanying Gaussian-distributed laser heat flux is applied on the top surface of each finite element. Thermal and structural mechanics are directly coupled, and the temperature field is applied as a thermal load to the subsequent structural analysis.

The numerical model of the stress field analysis involves meshing, material properties, boundary conditions, processing parameters, and computational scheme control. The mesh was refined for the cladding material to resolve large temperature and stress gradients. The minimum mesh size was set to 0.1 mm and the maximum mesh size was 0.4 mm. The computational domain was discretized using hexahedral meshes, generating a total of 30,263 meshes. The materials of the cladding material and the metal sheet were AlSi10 Mg, whose temperature-dependent material properties were referred from the ANSYS Workbench database.

The process parameters of the LC-based surface repair process were kept the same as those in the fluid flow analysis. The moving heat flux on the finite element was introduced using ANSYS APDL. The heat convection between the substrate surface and the gas was set for heat conduction analysis, and a fixed support was set at the bottom of the substrate for structural analysis. The initial temperature of the heat conduction calculation was 22 °C, 17 calculation load steps were used, one finite element was loaded in each load step, and the time of each step was 0.275 s considering the scan speed and element size. The last load step was set to 10 s, which is sufficient for cooling after the cladding is completed. A dynamic time step was used for the calculations, with an initial time step of 3 × 10^−3^ s, a minimum time step of 3 × 10^−4^ s, and a maximum time step of 3 × 10^−2^ s.

### 3.3. Results and Analysis

**Figure 8:**
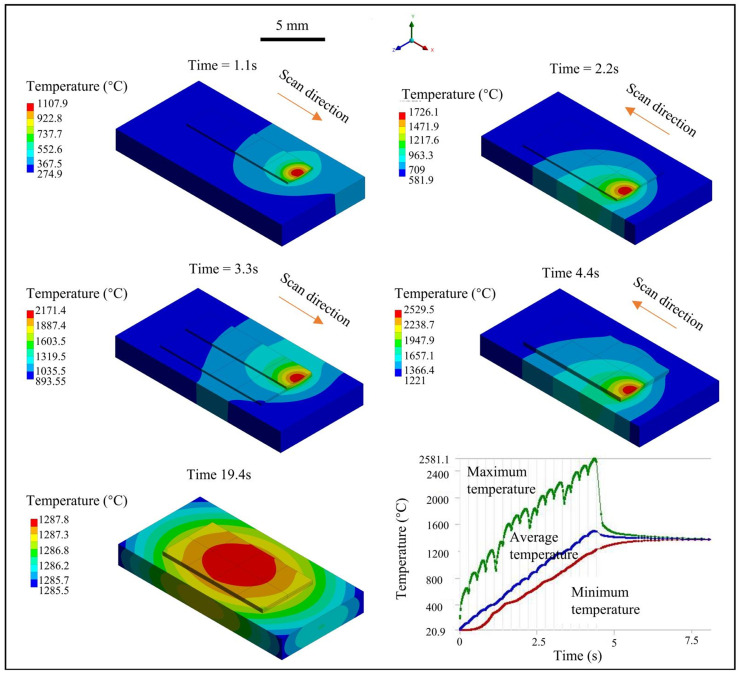
Temperature variation history during the multi-track and multi-layer LC-based surface repair process.

The temperature’s variation filed during the cladding process is shown in Figure 8. It indicates that the heat will gradually accumulate as the cladding layers are stacked layer by layer. The maximum temperature appears concentrated at the laser scanning position due to the Gaussian energy distribution character. When the 1-1 track is scanned, the temperature at the end of the melt track reaches above the material’s melting point, capable of forming a small molten pool, which is consistent with the results of the fluid flow simulation. For the 1-2 track, the maximum temperature of the material continues to rise, and a molten pool of sufficient size is formed to repair the surface flaw of the substrate. However, when the 2-1 and 2-2 tracks are scanned, the surface temperature continues to accumulate and increase, indicating that the molten pool continues to expand, and there is a risk of melting through the whole thin-walled substrates. The cooling process tends to be stable after cooling for 2.6 s, but it is difficult for the material to cool to room temperature. Therefore, for the multi-layer cladding of thin-walled components, the interval between layers should be more than 3 s to cool each cladding layer, and the cooling channel underneath the substrate is necessary to avoid the continuous expansion of the molten pool or melting through the components.

**Figure 9:**
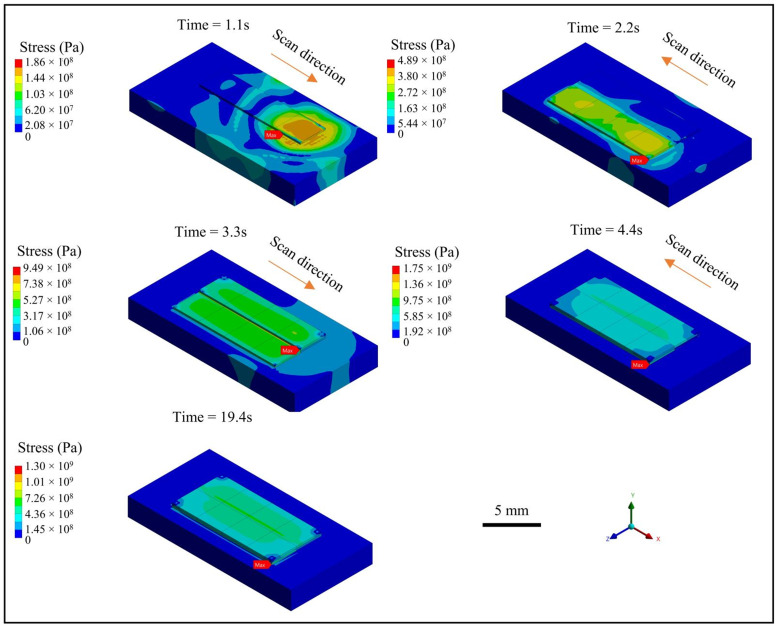
Stress variation history during the multi-track and multi-layer LC-based surface repair process.

The stress variation filed during the cladding process is shown in Figure 9. It shows that the maximum stress gradually increases with each track as well. When the 2-1 track is cladded, the maximum stress exceeds the yield strength of the aluminum alloy material, and plastic strain occurs, which will form residual stress after the material cools down. The maximum stress is mainly at the interfaces between the cladding layers, which can easily lead to interlayer cracking and cladding failure. Great compressive stress is also observed at the interface between tracks due to cyclic reheating, which alters the localized thermal gradient. The maximum stress is still at 1300 MPa, though the material is cooled down for 10 s after cladding, indicating a high risk of cracking at the corner between the substrate and the first layer. This finding is consistent with the experimental work by Bayat et al. [17], who observed pores and cracks in the first layer during their multi-track/multi-layer laser powder bed fusion process due to residual stress accumulation. Therefore, if the cladding layer is too thin, the temperature gradient between layers will be large, resulting in excessive intertrack and interlayer thermal stress and a risk of delamination.

## 4. Conclusions

The morphology, temperature, and velocity of the fluid flow of the molten pool during a multi-track LC-based surface repair process was investigated. All the mass, momentum, and energy due to powder feedstock were modeled and implemented during a finite volume numerical simulation. In addition, the stress and deformation fields during the multi-track and multi-layer LC-based surface repair process were studied using a coupled thermal–structural finite element numerical simulation. A multi-physical field numerical simulation of the molten pool was successfully executed. The key findings are summarized below.

(1) For effective defect repair, adjusting the laser power is crucial to ensure sufficient energy for melting the metal material and forming a stable molten pool. The molten pool presents a stable convection pattern, with a maximum flow rate of 0.2 m/s, a depth of 0.7 mm, and a width of 4 mm, ensuring that surface defects (300–900 μm) are completely repaired by multi-track cladding under the optimized parameters of 1600 W laser power and 600 mm/min scanning speed. The subsequent track can partially re-melt the prior adjacent track, facilitated by Marangoni convection in the molten pool, which has the potential to promote elemental redistribution and pore elimination at overlap regions. This study found that a 2 mm track spacing can achieve the best melt channel overlap effect and avoid surface unevenness for a beam diameter of 3.5 mm.

(2) Cyclic reheating during multi-track LC alters the localized thermal gradient, which induces compressive stresses at overlap boundaries and can contribute to the formation of cracks, affecting the overall cladding quality. The multi-layer of cladding results in an interlayer thermal stress peak of 1300 MPa, which exceeds the yield strength of aluminum alloys. An interlayer cooling interval of ≥3 s and an even cooling channel underneath the substrate are essential to reduce thermal stress during the LC process. Stress is concentrated at the interface between the cladding layer and the substrate, as well as at the overlap of the melt track. It is recommended to adopt a step-by-step power reduction strategy to reduce thermal gradients.

This simulation work directly addresses the critical industrial need to optimize laser cladding (LC) for the repair of aluminum alloy components, which are popular in the aerospace (e.g., turbine blades and structural joints) and automotive (e.g., engine blocks and transmission parts) industries due to their lightweight and high-strength properties. Surface damage to these high-value components often leads to complete replacement, incurring significant costs. Our model provides protentional and actionable insights to reduce such waste by quantifying optimal parameters and mitigating pores, residual stress, and cracking risks. Further investigations on physical phenomena such as material evaporation, pore formation, spatter, etc., under severe processing parameters are to be investigated. Direct multiphasic coupling to transfer the melt shape and temperature obtained in the flow model for stress modeling would be promising. Further studies on the micro-grain growth and grain boundary migration according to the melt temperature history obtained by flow modeling is necessary as well. The surface repair quality on substrates with different flaw shapes, sizes, and distributions should be further studied. Process optimization in terms of laser power, scanning speed, laser beam diameter, and layer thickness need to be performed using machine learning as well [25]. Direct experimental validation, such as the in situ high-speed X-ray imaging of molten pool behavior or residual stress measurements via neutron diffraction, will be essential to improve the fidelity of these simulation models.

## Figures and Tables

**Table 2:** Physical properties of argon applied in numerical simulation.

Name	Value
Density (kg/m^3^)	1.6288
Specific heat (J/(Kg K))	520.64
Heat conduction coefficient (W/(m K))	0.0158
Dynamic viscosity (Kg/(m s))	2.125 × 10^−5^
Molar mass (Kg/kmol)	39.948
Reference temperature (K)	298.15

## Data Availability

Due to privacy concerns, the ANSYS 2022 R1 Fluent UDF code presented in this study is only available on request from the corresponding author.
